# Building an integrated multidisciplinary care system for emergency surgery in abdominal cancer: a patient-centered perspective

**DOI:** 10.3389/fsurg.2026.1804666

**Published:** 2026-03-13

**Authors:** Qing Li, Jin-qiang Zhang

**Affiliations:** Department of General Surgery, The First Hospital of Yulin, Yulin, China

**Keywords:** abdominal cancer, emergency surgery, integrated care pathway, multidisciplinary collaboration, patient-centered care, surgical oncology

## Abstract

Abdominal oncologic emergencies demand urgent surgical decisions that may conflict with ongoing cancer therapies and patient preferences. Conventional fragmented care models often lead to delays, inconsistent decisions, and poor care transitions. To address this gap, this perspective article proposes an integrated, patient-centered care model structured in three stages: rapid initial assessment, a structured emergency multidisciplinary team meeting, and coordinated care with seamless transitions. Central to this framework is a structured decision-making guide for emergency surgery that comprehensively integrates patient factors (health status, prognosis), tumor-related factors (emergency severity, technical feasibility), and patient preferences and context. Based on this triad, clinicians can pursue one of three clear pathways: immediate stabilization, definitive emergency oncologic surgery for suitable candidates, or prioritized non-operative or palliative-first approach when surgery offers limited benefit. Supported by innovations like standardized pathways and emergency-specific performance metrics, this framework aims to enhance safety, timely goal-concordant care, and resource efficiency in abdominal cancer emergency surgery.

## 1 Introduction

Abdominal oncologic emergencies are acute, life-threatening conditions caused by primary or metastatic abdominal tumors or their treatment (1, 2). These include serious surgical and medical events such as bowel obstruction, severe hemorrhage, organ failure due to tumor mass effect, and metabolic crises (2–4). Managing these emergencies is particularly challenging due to their sudden onset, rapid progression, and diagnostic complexity (3, 5). An immediate conflict often arises between addressing the urgent complication and managing the underlying cancer, such as when surgery is needed for a patient with recent chemotherapy-induced immunosuppression.

Traditional care models, which operate in specialty silos like emergency medicine, surgical oncology, and medical oncology, often worsen these challenges (6). This fragmented approach can lead to delayed diagnosis, inconsistent treatment plans, poor coordination, and a disjointed experience for patients who are passed between departments (7, 8).

A patient-centered framework is essential to overcome these limitations. This approach focuses not only on the acute medical issue but also integrates the patient's overall health, treatment history, personal values, and psychosocial context into a unified care plan (9, 10). Theoretical models from complex systems science and collaborative healthcare support that such intricate clinical situations require a structured, multi-specialty strategy (9). Therefore, an institutionalized multidisciplinary team (MDT) model is fundamental. It ensures early involvement of all relevant specialists, facilitates simultaneous assessment, promotes consensus on treatment priorities, and maintains clear communication. Establishing this comprehensive collaborative approach is critical for developing an effective, efficient, and patient-focused system for managing abdominal oncologic emergencies.

## 2 Core pillar: the structure and role definition of a MDT

### 2.1 Core members and extended support network

The effective management of abdominal oncologic emergencies requires a well-defined MDT (11). This team integrates essential core specialists with an extended network of supporting disciplines. The core members include surgical oncology, medical oncology, interventional radiology, critical care, and emergency medicine (11, 12). These specialists provide direct intervention for acute diagnostic and therapeutic challenges. Timely contributions from pathology and radiology are also crucial for accurate initial diagnosis and staging (13).

An extended support network is vital for addressing comprehensive patient needs. This network encompasses specialists in anesthesiology, pain and palliative care, clinical nutrition, mental health, specialized nursing, clinical pharmacy, and social work (14). Their involvement ensures holistic attention to comorbidities, treatment side effects, nutritional status, psychological well-being, and social barriers (15). This comprehensive approach is essential for informed decision-making and patient-centered outcomes.

### 2.2 Role redefinition and collaborative synergy

Successful collaboration necessitates a shift from episodic consultation to integrated care (16). Each member should become a co-responsible stakeholder in a unified treatment plan, moving beyond traditional specialty boundaries (17). Clear role definition within this framework enhances efficiency. For example, the medical oncologist contributes knowledge of systemic disease and treatment history. The surgical oncologist evaluates the necessity and risks of emergency surgery. The interventional radiologist provides minimally invasive procedural options (18). Concurrently, the palliative care specialist manages complex symptoms and facilitates communication about treatment goals (19). This synergy enables the parallel management of the acute emergency and the underlying cancer, fostering a cohesive and personalized care strategy.

## 3 System architecture: An integrated pathway centered on the patient journey

**Figure 1:**
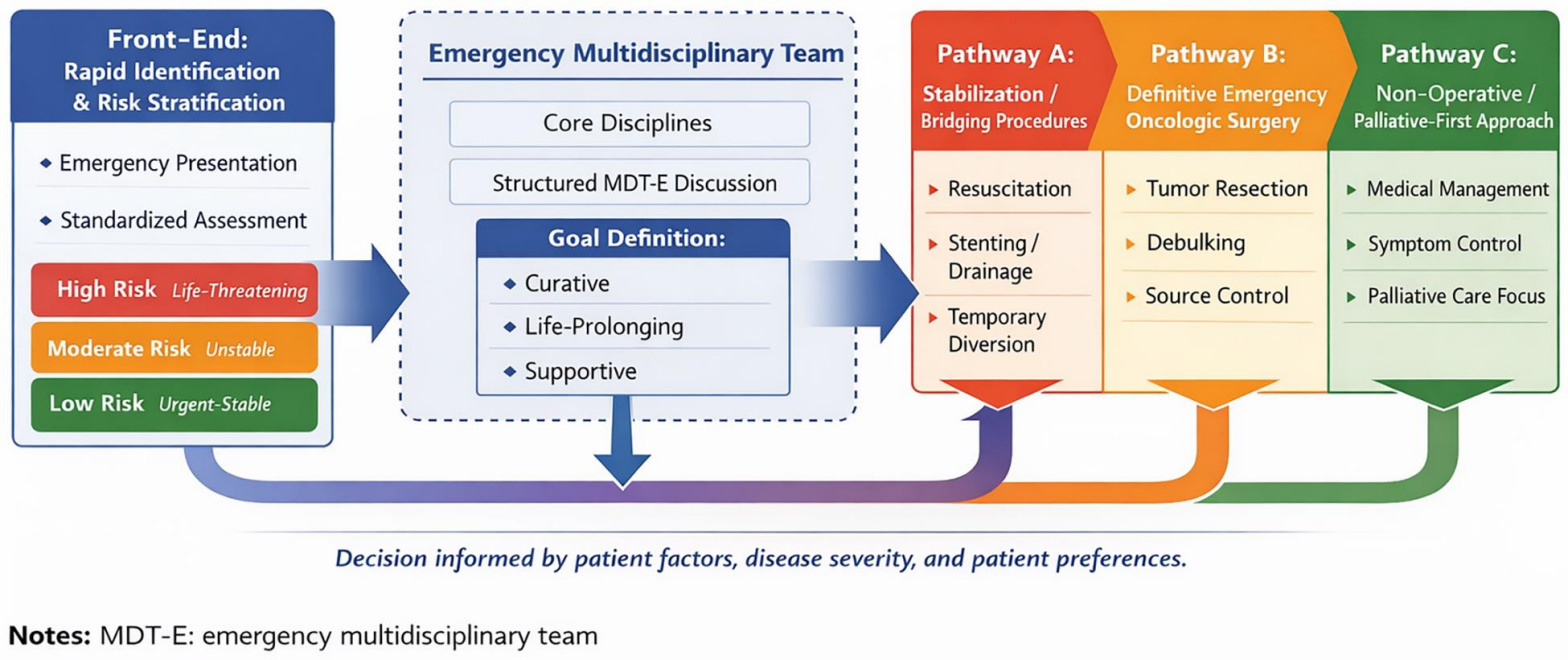
Integrated patient-centered pathway for emergency surgery in abdominal cancer. MDT-E, emergency multidisciplinary team.

This system architecture is organized around the patient's journey, creating an integrated clinical pathway (Figure 1). It comprises three key stages: rapid front-end identification, structured mid-end decision-making, and coordinated back-end treatment. As illustrated in Figure 1, the front-end identification stage serves as the entry point to the integrated emergency oncology pathway. This visual framework highlights the dynamic progression from initial triage and risk stratification to structured multidisciplinary deliberation and subsequent definitive intervention. This staged configuration ensures temporal continuity, clinical coherence, and goal alignment throughout the emergency care process.

### 3.1 Front-End: rapid identification and risk stratification channel

The front-end focuses on rapid identification and risk stratification. A proactive “alert-response” mechanism involves educating patients and caregivers about critical symptoms during routine oncology visits, with emphasis on recognizing high-risk symptoms suggestive of acute oncologic complications (20). This anticipatory strategy promotes early presentation and reduces delays in accessing emergency care. Upon arrival at the emergency department, a standardized assessment protocol is activated. This protocol integrates rapid tumor-specific laboratory testing, prioritized imaging, and structured clinical evaluation (21). The process allows for immediate risk stratification into categories such as *immediately life-threatening, potentially deteriorating*, or *stable but urgent*, which then guides the subsequent pace and level of intervention (22). This stratification directly determines the urgency, intensity, and composition of subsequent interventions. By formalizing the transition from triage to escalation, the front-end stage establishes clinical stability as a prerequisite for higher-level multidisciplinary deliberation.

### 3.2 Mid-End: structured multidisciplinary emergency discussion

The mid-end centers on a structured MDT meeting for emergencies. This team operates through both scheduled rapid briefings and immediate activation pathways for urgent cases (23). The purpose is to consolidate complex information into a unified decision-making process under time constraints. To ensure efficiency and reproducibility, a standardized information template is employed. This template consolidates essential variables, including medical history, tumor characteristics, imaging findings, laboratory abnormalities, performance status, and patient preferences (24, 25). Such structured data synthesis minimizes cognitive fragmentation and enhances decision transparency. Decision-making follows a goal-oriented framework. The MDT first establishes the primary therapeutic intent-curative, life-prolonging, or supportive-before determining the appropriate sequence of interventions (25). This alignment ensures that acute stabilization strategies are integrated with broader oncologic objectives rather than considered in isolation. The resulting consensus produces a coordinated, staged treatment plan, such as prioritizing urgent stabilization before reassessing eligibility for definitive anti-tumor therapy (26).

### 3.3 Emergency surgery for abdominal cancer: A decision-making framework

Emergency surgical decision-making in abdominal cancer is inherently complex and time-sensitive. It requires careful reconciliation of oncologic intent, physiological reserve, and the severity of the acute complication. Unlike elective surgical planning, emergency scenarios allow limited time for deliberation and demand a structured yet adaptable analytical framework capable of supporting rapid, individualized, and ethically grounded decisions.

To address this clinical need, we propose a tripartite assessment model that organizes clinical reasoning into three interrelated domains. As outlined in Table 1, these domains translate conceptual considerations into practical decision-making criteria. In clinical practice, this model is operationalized through a sequential stepwise approach. First, clinicians conduct a rapid assessment of the patient's physiological reserve and frailty. Second, they evaluate the technical feasibility of the surgery and clarify the oncologic intent. Third, they engage in discussions to ascertain the patient's treatment goals and anticipated quality-of-life outcomes. This structured sequence transforms the conceptual framework into an actionable bedside tool, facilitating real-time clinical decision-making. The table operationalizes the framework by mapping each domain to clinically observable indicators, thereby serving not as a static summary, but as a structured bedside checklist for real-time evaluation during emergency MDT discussions. This design promotes consistency, transparency, and reproducibility in high-stakes clinical decision-making (23).

**Table 1:** Decision-making framework for emergency surgery in abdominal cancer.

Decision domain	Core assessment	Implication for emergency decision-making	Preferred strategy
Patient factors	Functional status, frailty, comorbidities, prognosis	Determines physiological tolerance and likelihood of meaningful recovery	Stabilization/bridging vs. selective definitive surgery
Disease and emergency-related factors	Tumor stage/resectability; type and severity of emergency	Defines urgency and technical feasibility of surgery	Damage-control, definitive emergency surgery, or staged approach
Contextual and preference-based factors	Goals of care, expected quality of life, resources	Ensures goal-concordant and ethically appropriate care	Non-operative or palliative-first when benefit is limited
Integrated MDT-E synthesis	Multidisciplinary consensus and dynamic reassessment	Translates multidimensional inputs into a unified plan	Adaptive pathway with reassessment after stabilization

MDT-E, emergency multidisciplinary team.

The first domain encompasses patient-related factors, such as functional status, frailty, comorbidities, and overall prognosis (27, 28). The second domain includes disease- and emergency-related factors, namely tumor stage, resectability, and the nature and severity of the acute event—for instance, obstruction, perforation, hemorrhage, or sepsis (29). The third domain integrates contextual and preference-based considerations, including the patient's goals of care, anticipated quality of life, and available institutional resources (30). This comprehensive evaluation supports the stratification of patients into distinct management pathways.

Based on this stratification, emergency surgical strategies can be categorized into three main types. The first consists of damage-control or bridging procedures, aimed at rapid stabilization and symptom relief, such as diversion, decompression, or emergency interventional radiology (31). The second is definitive oncologic surgery performed during the acute phase, which is reserved for selected patients with sufficient physiological reserve and a clear expectation of survival benefit (32). The third comprises non-operative or palliative-first approaches, focusing on symptom control and goal-concordant care when surgical risk is considered too high (33). This framework highlights the necessity of individualized management over rigid protocols in addressing abdominal oncologic emergencies.

To further demonstrate the practical application of this decision-making framework, two clinically representative hypothetical scenarios are outlined below.

#### 3.3.1 Case A (definitive emergency surgery candidate)

A 62-year-old male with locally advanced but potentially resectable sigmoid colon cancer presents with acute large-bowel obstruction. He has an Eastern Cooperative Oncology Group (ECOG) performance status of 1, well-controlled hypertension, and no evidence of distant metastasis. Imaging confirms obstruction without perforation. Following structured MDT discussion integrating physiological reserve, tumor resectability, and the patient's preference for active treatment, emergency oncologic resection is undertaken. This course of action corresponds to the definitive emergency surgery pathway derived from the tripartite assessment model.

#### 3.3.2 Case B (damage-control/palliative-oriented strategy)

A 78-year-old female with metastatic gastric cancer undergoing second-line chemotherapy presents with perforation and septic instability. She has an ECOG performance status of 3 and marked frailty. MDT evaluation identifies limited oncologic benefit from radical surgery and substantial perioperative risk. After explicit goals-of-care discussion, the team selects a damage-control procedure combined with symptom-directed management and early palliative integration rather than curative resection.

These illustrative cases demonstrate how structured evaluation across patient-related, disease-related, and contextual domains enables differentiated yet coherent emergency surgical strategies. They further emphasize that the framework operates as a dynamic decision-support structure rather than a prescriptive algorithm, thereby preserving clinical judgment while enhancing decision clarity under time constraints.

### 3.4 Back-End: integrated treatment and seamless transitions

The back-end ensures integrated treatment and seamless transitions. A co-management model is adopted, with a designated lead physician coordinating the overall plan while various specialists execute their roles concurrently (34). Meticulous transition protocols are critical for safe patient transfer between departments, maintaining continuity of information and care (35). Furthermore, palliative and supportive care are integrated from the outset (36, 37). Specialists in this field engage early to manage symptoms, address psychological needs, and facilitate discussions about care goals, ensuring patient values remain central throughout the treatment process.

### 3.5 Surgical pathways within the emergency multidisciplinary framework

Effective emergency surgery for abdominal cancer requires a distinct MDT structure, adapted for urgent decision-making (38). This emergency MDT model prioritizes rapid team assembly, time-sensitive deliberation, and defined surgical leadership to meet the demands of acute clinical scenarios.

Within this framework, the emergency surgeon coordinates inputs from relevant specialists, including surgical and medical oncology, anesthesiology, critical care, interventional radiology, and palliative care (39). Unlike elective care discussions, the emergency MDT focuses primarily on immediate risk reduction, procedural feasibility, and perioperative stability, rather than long-term treatment sequencing alone (40). This prioritization acknowledges that delays or undue complexity in decision-making can adversely affect clinically unstable patients.

A central feature of this model is its capacity for iterative reassessment. An initial stabilization procedure may be followed by re-evaluation for definitive cancer-directed surgery once the patient's condition allows (11). Conversely, early identification of excessive surgical risk or limited benefit permits a timely shift to non-operative or palliative strategies (11). Incorporating these responsive pathways into the emergency MDT process promotes both clinical efficiency and transparent, goal-concordant care in abdominal cancer emergencies.

### 3.6 Innovations in emergency surgery for abdominal cancer

Innovations in emergency surgery for abdominal cancer are advancing on three interconnected fronts, reflecting a systemic shift from isolated surgical interventions toward integrated care systems. This evolution prioritizes comprehensive process and decision-support innovations over mere technological upgrades to enhance the safety and effectiveness of emergency management.

First, standardized emergency oncology pathways are being established to enable rapid multidisciplinary collaboration through clear activation criteria and time-sensitive protocols for critical complications (41). These pathways, along with structured real-time multidisciplinary consultation and digital decision aids, collectively aim to accelerate intervention, reduce unnecessary high-risk surgeries, and better align urgent surgical needs with long-term oncologic goals (40).

Second, the integration of minimally invasive techniques (such as laparoscopic diversion and endoscopic stenting) and digital tools—including the aforementioned decision aids as well as real-time clinical dashboards—enhances both procedural options and care coordination (42, 43). Designed to be scalable and measurable, these innovations facilitate systematic quality improvement.

Third, outcome measurement is being refined by incorporating emergency-specific indicators into continuous quality improvement cycles (41). This includes tracking key performance indicators such as decision-making time, treatment-goal alignment, and patient outcomes, alongside traditional metrics like time to intervention and postoperative morbidity (41, 44). Collectively, these developments ensure that advancements are systematically evaluated and contribute to an overarching framework focused on measurable, patient-centered results.

## 4 Enabling and support: Key elements of the infrastructure

### 4.1 Physical and digital infrastructure development

A strong infrastructure is essential for implementing the integrated care pathway. This requires a dedicated physical space, such as a designated oncology emergency care unit (45, 46). This unit should be designed for rapid assessment, stabilization, and time-sensitive treatment, which improves workflow and minimizes delays (45).

An advanced digital platform is equally critical. This platform should connect key systems like electronic health records, medical imaging archives, and lab data into one unified interface (47). It should also include tools for team collaboration, such as secure messaging and shared decision templates, allowing real-time data access for all members (48). This integration supports joint planning and ensures all providers share the same current clinical information.

### 4.2 Institutional and cultural foundation

Effective long-term collaboration depends on both formal systems and a supportive environment. Institutions should establish clear written protocols, including a collaboration charter that defines roles and emergency procedures (49). Leadership can be shared through a rotating system among core specialties to promote collective responsibility (50). Additionally, using shared performance goals focused on patient outcomes—such as time to treatment—helps align the team's efforts.

Cultivating the right team culture is also vital (51). This means creating an atmosphere of mutual respect and trust where every specialty's input is valued. Leaders should actively promote this culture, encouraging teams to move beyond traditional boundaries (52). The focus should shift from protecting departmental autonomy to prioritizing coordinated, high-quality patient care.

### 4.3 Education and continuous training

The system's success relies on the skills and teamwork of its staff. Targeted education programs are therefore necessary to enhance clinical competencies in managing abdominal cancer emergencies, including diagnosis, urgent intervention, and supportive care (53).

Training should also address the collaboration process itself (54). Regular multidisciplinary simulation exercises are crucial. These drills should mimic high-pressure emergency scenarios, allowing teams to practice communication, clarify roles, make group decisions quickly, and resolve disagreements in a safe setting (55). Ongoing training of this kind reinforces procedures, builds team cohesion, and prepares staff to work together effectively during actual emergencies.

## 5 Evaluation and continuous improvement: metrics for system success

The effectiveness and sustainability of the integrated oncology emergency care system rely on a comprehensive evaluation framework. This framework should assess progress in three key areas: direct patient outcomes, internal system efficiency, and the capacity for ongoing refinement.

### 5.1 Patient-Centered outcome metrics

Success is best measured by improvements in patient welfare. Evaluation should focus on meaningful clinical endpoints that reflect care quality and timeliness. Important process measures include critical time intervals, such as the period from emergency department arrival to definitive treatment (56). For abdominal oncologic emergencies specifically, two intervals warrant precise monitoring: the time from patient presentation to a treatment decision, and the time from that decision to the actual intervention (56). Key outcome measures track complication rates—particularly major postoperative complications—the need for additional surgery, 30-day unplanned readmission rates, and relevant survival data, including short-term mortality (e.g., within 30 or 90 days) (57, 58). Intensive care unit admission rates serve as another vital indicator of resource utilization and postoperative severity. Within a coordinated oncology emergency system, tracking these outcomes facilitates a precise assessment of whether the urgent surgical intervention was appropriate, safe, and valuable (58). Furthermore, it is essential to incorporate the patient's own perspective. This can be achieved through standardized patient-reported outcome measures, which assess symptom control, quality of life, and satisfaction with care received (59). These combined measures ensure that treatment processes and goals align with both clinical efficacy and what patients value most.

### 5.2 System performance and process metrics

To ensure the multidisciplinary model operates efficiently, specific process indicators are necessary (60). These metrics monitor the functionality of the collaboration itself. They include the operational efficiency of the MDT, measured by the time from patient referral to team discussion and the rate at which team decisions are carried out (61). Additional metrics should evaluate care coordination, such as transfer times between departments, and the appropriate use of critical resources (60). Tracking these indicators helps identify bottlenecks and inefficiencies within the clinical pathway (62).

### 5.3 Cycle of continuous quality improvement

Data collection serves as the starting point for systematic improvement, not the final goal. A structured continuous quality improvement cycle, such as the Plan-Do-Study-Act model, should be formally adopted (63, 64). This involves regularly collecting and analyzing data from both outcome and process metrics. The resulting insights help pinpoint specific areas for improvement, such as streamlining a referral process. Proposed changes are then planned, tested on a small scale, evaluated for their impact, and—if successful—integrated into standard practice (63, 65). This ongoing, data-driven cycle ensures the care system remains adaptable, responsive to evidence, and consistently focused on enhancing both patient results and operational performance (65, 66).

## 6 Discussion and future perspectives

### 6.1 Summary and core arguments

The proposed framework systematically addresses the complex challenges of abdominal cancer emergencies. It replaces a fragmented, department-focused approach with an integrated, patient-centered model (67). This system establishes structured MDT, standardized pathways, and essential support structures (68). It tackles critical problems like delayed diagnosis, inconsistent treatments, and poor coordination. The central thesis is that holistic care—addressing medical, psychological, and social needs—requires formal collaborative systems (38, 69).

While components such as MDT involvement and emergency surgery protocols are not novel in isolation, the innovation of this framework lies in their systematic integration into a unified, time-sensitive, and goal-concordant architecture specifically designed for abdominal oncologic emergencies. This structural synthesis distinguishes the model from broader multidisciplinary recommendations by embedding these elements into a cohesive system that aligns urgent intervention with long-term cancer management, thereby aiming to improve both immediate clinical outcomes and the overall patient journey.

This article presents a conceptual and structural framework rather than an empirical validation study. Its primary contribution lies in proposing a clinically applicable and system-oriented architecture for emergency oncologic care. Although the framework is grounded in existing evidence and multidisciplinary principles, its practical effectiveness requires empirical confirmation. Therefore, prospective implementation studies are necessary to evaluate its impact in real-world clinical settings.

### 6.2 Addressing implementation challenges

Implementing this model will encounter practical obstacles. Major challenges include coordinating multiple departments administratively, covering the initial costs for space and technology, and redesigning performance metrics to encourage teamwork (70). The most significant hurdle may be changing established professional cultures and breaking down traditional departmental barriers (71). Initial strategies to overcome these include gaining strong leadership support, starting with a controlled pilot program, creating shared accountability measures, and investing in ongoing team training (72). Proactively managing these organizational and human factors is vital for successful implementation (72).

### 6.3 Future research directions

This framework opens several important avenues for future investigation, spanning methodological validation, technological innovation, and cross-disciplinary adaptation. To ensure both conceptual advancement and practical applicability, subsequent research should proceed along structured and empirically rigorous pathways.

Future research should empirically validate the proposed decision-making framework through several rigorous study designs. While the present article advances a system-level architecture for emergency oncologic care, its clinical utility should be demonstrated through methodologically sound evaluation. First, prospective multicenter cohort studies are needed to compare clinical outcomes before and after implementation of the structured pathway (73). Such designs would allow assessment of temporal changes in safety, timeliness, and goal concordance under real-world conditions. Second, cluster-randomized trials should evaluate whether MDT-guided emergency care, when compared with conventional silo-based decision-making, leads to improved patient outcomes (74). This approach would strengthen causal inference by reducing contamination between intervention and control strategies. Third, comparative effectiveness research should assess key performance metrics, including time-to-decision, complication rates, goal-concordant care, and cost-effectiveness (75, 76). By integrating clinical, operational, and economic indicators, these studies would provide multidimensional evaluation of system performance. Together, these investigations would provide robust empirical validation of the proposed system architecture and its clinical utility.

Beyond validation, methodological refinement of decision-support tools remains a priority. First, developing specialized clinical tools is critical, such as models to predict patient risk earlier and digital systems to support team decision-making in urgent cases (77). Such tools may incorporate machine learning–based prognostic algorithms or real-time dashboards to enhance data synthesis during emergency consultations. Second, researchers should explore the role of technology, including studying how telehealth can effectively connect remote specialists to urgent team meetings (78). Evaluating the feasibility, reliability, and governance implications of these technologies will be critical for scalable implementation. Third, broader comparative studies are required to measure the overall value of this integrated model relative to standard care pathways (79). These investigations should incorporate outcome measures spanning mortality, morbidity, resource utilization, patient-reported outcomes, and institutional efficiency. Health economic analyses will be particularly important in determining long-term sustainability. Finally, studies should examine whether this integrated approach can be effectively adapted for other acute cancer complications, such as those in neurology or thoracic oncology (80).

Collectively, these research directions aim not only to validate the framework but also to refine, expand, and adapt it within evolving clinical ecosystems. Rigorous empirical testing, technological integration, and cross-contextual application will be essential for transforming this conceptual model into a reproducible and evidence-based standard of emergency oncologic care.

## 7 Summary

Establishing a patient-centered, integrated care system for abdominal cancer emergencies is essential for managing the increasing complexity of emergency surgical decision-making in oncologic patients. This framework replaces fragmented, specialty-driven practices with a unified, goal-focused treatment network. It supports timely risk assessment, structured multidisciplinary collaboration in emergencies, and coordinated care that aligns with the patient's prognosis and preferences.

Importantly, this approach redefines emergency cancer surgery. It is not an isolated technical procedure, but a context-aware clinical strategy. This strategy operates within a framework of team-based oversight, quality monitoring, and ongoing refinement. Using standardized pathways, clear decision tools, and specific performance measures, the system tackles key issues in safety, treatment appropriateness, and alignment with patient goals.

Progressing this integrated model demands sustained cooperation among surgeons, oncologists, hospital leaders, and policymakers. Their collaboration is necessary to implement, evaluate, and scale the system. These efforts are crucial to enhance clinical outcomes while respecting the dignity and personal values of critically ill cancer patients during emergency surgical care.

## Data Availability

The original contributions presented in the study are included in the article/Supplementary Material, further inquiries can be directed to the corresponding author.
